# Clinical and Prognostic Implications of Estimating Glomerular Filtration Rate by Three Different Creatinine-Based Equations in Older Nursing Home Residents

**DOI:** 10.3389/fmed.2022.870835

**Published:** 2022-04-26

**Authors:** Ersilia Paparazzo, Silvana Geracitano, Vincenzo Lagani, Luca Soraci, Annalisa Cozza, Salvatore Cosimo, Francesco Morelli, Andrea Corsonello, Giuseppe Passarino, Alberto Montesanto

**Affiliations:** ^1^Department of Biology, Ecology and Earth Sciences, University of Calabria, Rende, Italy; ^2^Institute of Chemical Biology, Ilia State University, Tbilisi, Georgia; ^3^Biological and Environmental Sciences and Engineering Division (BESE), King Abdullah University of Science and Technology (KAUST), Thuwal, Saudi Arabia; ^4^Unit of Geriatric Medicine, Italian National Research Center on Aging (IRCCS INRCA), Cosenza, Italy; ^5^Laboratory of Pharmacoepidemiology and Biostatistics, Italian National Research Center on Aging (IRCCS INRCA), Cosenza, Italy; ^6^SADEL S.p.A., Cotronei, Italy

**Keywords:** chronic kidney disease (CKD), Berlin initiative study (BIS), full age spectrum (FAS), estimated glomerular filtration rate (eGFR), older patients

## Abstract

**Background:**

According to the international literature, the percentage of nursing home (NH) residents with renal insufficiency is very high, ranging between 22 and 78%. Diminished kidney function represents a risk factor for drug overdosage, adverse drug reactions, end-stage renal disease, disability, morbidity, and mortality. Several studies suggested that screening for chronic kidney disease (CKD) in high-risk and older populations may represent a cost-effective approach to reducing progression to renal failure and CKD mortality.

**Objective:**

This study aimed (i) to investigate to what extent CKD may be staged interchangeably by three different creatinine-based estimated glomerular filtration rate (eGFR) equations in a sample of older adults living in long-term care facilities; (ii) to investigate factors explaining differences among eGFR equations; and (iii) to compare the predictivity of different creatinine-based eGFR equations with respect to all-cause mortality.

**Methods:**

A total of 522 residents aged 65 years and older participated in a prospective cohort study of 9 long-term care facilities in Calabria. eGFR was calculated by Chronic Kidney Disease Epidemiology Collaboration (CKD-EPI), Berlin initiative study (BIS), and full age spectrum (FAS) equations. Disability in at least one activity of daily living (ADL), depression, cognitive impairment, comorbidity, and malnutrition was considered in the analysis. Statistical analysis was carried out by Bland–Altman analysis, and 2-year mortality was investigated by Kaplan–Meier curves and Cox regression analysis.

**Results:**

Depending on the adopted equation, the prevalence of NH residents with impaired renal function (eGFR < 60 ml/min/1.73 m^2^) ranged between 58.2% for the CKD-EPI and 79.1% for the BIS1 equation. The average difference between BIS and FAS was nearly negligible (0.45 ml/min/1.73 m^2^), while a significant bias was detected between CKD-EPI and BIS and also between CKD-EPI and FAS (6.21 ml/min/1.73 m^2^ and 6.65 ml/min/1.73 m^2^, respectively). Although the eGFR study equations had comparable prognostic accuracy in terms of mortality risk, BIS and FAS were able to reclassify NH residents pertaining to a low-risk group with CKD-EPI, and this reclassification improves the discriminative capacity of CKD-EPI with respect to overall mortality.

**Conclusion:**

Despite the relatively good correlation between eGFRs calculated using all adopted equations, the findings in this study reported clearly demonstrated that CKD-EPI and BIS/FAS equations are not interchangeable to assess eGFR among older people and particularly in institutionalized and frail older subjects.

## Introduction

According to the international literature, the percentage of nursing home (NH) residents with renal insufficiency is very high, ranging between 22 and 78% (1–7). Assessment of renal function is particularly important in elderly patients for which diminished kidney function represents a risk factor for drug overdosage and adverse drug reactions (ADRs) (8), end-stage renal disease (ESRD) (9), cardiovascular diseases (CVD) (9–12), disability, morbidity (13), and mortality (9, 14). Chronic kidney disease (CKD) is particularly harmful because many affected patients are initially asymptomatic and may remain undiagnosed for several years. For this reason, screening measures for the early identification of high-risk subjects in older populations would be of help, and several national, European, and international initiatives have been carried out in recent years to this end (14–20). In fact, several studies suggested that screening for CKD in older populations such as institutionalized elderly people may represent a cost-effective approach to reducing progression to renal failure and CKD mortality (21, 22). Preventing/slowing CKD progression among these patients may substantially impact several social and health domains, e.g., decreasing the need for long-term assistance and the healthcare costs related to caregiving (23).

Estimated glomerular filtration rate (eGFR) equations are routinely used for the clinical assessment of kidney function. Since reliable gold-standard methods for measuring GFR are too complicated and not always easily suitable in clinical practice, it is usually assessed by simple creatinine-based equations (22). Although the eGFR calculated from serum creatinine is widely used in general practice, the age-related loss of muscle mass that affects 40–85% of NH residents (23) together with the age-related decline in protein intake can maintain serum creatinine at a normal level in spite of reduced kidney function. This causes an overestimation of the GFR making serum creatinine a poorly reliable marker for estimating GFR and, consequently, CKD prevalence. In this context, it is not surprising that several studies showed the existence of a U-shaped relationship between creatinine-based eGFR and mortality in elderly people (24–28). To overcome this important limitation, two equations specifically developed for older populations were proposed, namely, the Berlin initiative study (BIS) (29) and the full age spectrum (FAS) equation (30). Although more suitable for older subjects, due to non-GFR determinants of serum creatinine, these new equations still provide a not negligible bias in the assessment of the GFR in this population segment.

Studies including age-specific equations and investigating agreement among different eGFR equations of their ability to predict long-term prognosis in older nursing home residents are distinctively lacking. Therefore, this study aimed (i) to investigate to what extent CKD may be staged interchangeably by three different creatinine-based eGFR equations in a sample of older adults living in long-term care facilities; (ii) to investigate factors explaining differences among eGFR equations; and (iii) to compare the predictivity of different creatinine-based eGFR equations in regards to overall mortality.

## Materials and Methods

### Sample

Patients consecutively admitted to nine participating nursing homes (NHs) during the 24 months were asked to participate in this study. A total of 726 NH residents aged 65 years and older were initially screened and enrolled. All recruited subjects underwent a multidimensional geriatric assessment with detailed clinical history, including anthropometric measures and a set of the most common tests to assess cognitive functioning, functional activity, physical performance, nutritional status, and depression. In addition, common clinical hematological tests were performed. For the current analysis, NH residents with missing mortality data (*n* = 176) or missing values for serum creatine (*n* = 28) were excluded, leaving 522 patients to be included in the analyses regarding the agreement between equations and survival analysis. Informed written consent was signed by all subjects or their legal representatives. This study protocol was approved by the Regional Ethical Committee, Catanzaro, Italy (Prot. CE 119/2016).

### Outcomes

The main outcomes of the present study were agreement among eGFR equations and overall mortality. Patients were followed-up every 12 months for 2 years to collect information about their vital status. In the case of discharged patients, this information was collected by telephone call during which patients and/or their relatives/caregivers were interviewed. For patients who died during the follow-up period, information about the date, place, and cause of death was collected from death certificates provided by relatives or caregivers.

### Comprehensive Geriatric Assessment

The management of activities of daily living (ADL, i.e., bathing, dressing, eating, and independence in and out of bed) was assessed by means of a comprehensive geriatric assessment using a modification of an international and widely used scale, the Katz’ Index of ADL (31). The assessment was based on activities the subject was able to perform at the time of the visit. Each activity was scored as 0 if patients were unable to perform the task and 1 for people able to perform such activity. Then, ADL scores ranged between 0 (unable to perform any activity) and 5 (able to perform all the activities).

Cognitive status was assessed using the age- and education-adjusted Mini-Mental State Examination (MMSE) score (32), and patients scoring less than 24 were considered cognitively impaired. Depression was defined as having a 15-item Geriatric Depression Scale (GDS) score > 5 (33).

Nutritional status was performed using the Mini Nutritional Assessment (MNA), a well-validated tool for assessing malnutrition in old people (34). MNA includes 18 self-reported questions derived from general, anthropometric, dietary, and self-assessment. In particular, the short form of the MNA (MNA-SF) is a screening tool consisting of six questions on food intake, weight loss, mobility, psychological stress, acute disease, the presence of dementia or depression, and body mass index (BMI). The maximum score for the MNA-SF is 14. A score lower than 12 points implicates the presence of malnutrition/malnutrition risk (23). Hypertension, heart failure (HF), diabetes, cancer, coronary artery disease (CAD), cerebrovascular diseases (CVD), and chronic obstructive pulmonary disease (COPD) were also considered in the analyses. Overall comorbidity was assessed by the Cumulative Illness Rating Score for Geriatrics (CIRS-G) (35). CIRS-G evaluates the severity of coexisting diseases in 14 organ/system scales, each ranging from 0 (problem absent) to 4 (severe problem with the requirement of the immediate treatment and/or severe organ/system failure). A sum score ranging from 0 to 56 points was then calculated by adding each system/organ score. In the case of multiple diseases affecting one organ/system, only the most severe condition was considered in the calculation of the CIRS-G score.

### Measurements of Serum Creatinine

Recruited subjects underwent a blood sampling after overnight fasting for general laboratory screening at the time of enrollment. Serum samples were immediately stored at −80^°^C until assayed. The general laboratory panel included serum creatinine measured using the standardized Jaffe method calibrated to isotope dilution mass spectrometry using the automated analyzer (RX-30, Nihon Denshi Inc., Tokyo, Japan).

### Estimated Glomerular Filtration Rate Equations

The GFR was estimated by the creatinine-based Chronic Kidney Disease Epidemiology Collaboration (CKD-EPI) (36), FAS (30), and BIS1 (29) equations:

CDK-EPI = 141 × min (S_Cr_,κ)^α^ × max (S_Cr_,κ)^1.209^ × 0.993 ^age^ [ × 1.018 if women],

where κ = 0.7 for women and 0.9 for men; α = −0.329 for women and −0.411 for women; min indicates the minimum of SCr/κ or 1; and max indicates the maximum of SCr/κ or 1.

FAS = 107.3/[S_Cr_/Q] [× 0.988^(age–40)^ when age > 40 years], with Q = 0.70 for women and 0.90 for men.

BIS1 = 3,736 × S_Cr_^–0.87^ × age^–0.95^ [× 0.82 if women].

Patients were grouped according to kidney function as follows: eGFR > 60, 45–59.9, 30–44.9, and < 30 ml/min/1.73 m^2^.

### Analytic Procedure

First, crude correlations among glomerular filtration rates calculated by CKD-EPI, BIS, and FAS equations were graphically investigated. Bland–Altman plots were generated to analyze the difference between CKD-EPI-BIS, CKD-EPI-FAS, and BIS-FAS against the mean of the two estimates, respectively, on the whole cohort of NH residents. Then, we compared the distribution of NH residents into CKD stages based on eGFRs calculated using the adopted equations.

Second, to identify factors explaining the difference between the different eGFR estimation formulas, we used the following three-step procedure: (a) linear regression analysis was used to select variables associated in a univariate way with the difference between two eGFR estimation methods; (b) variables selected by the univariate approach were entered into a stepwise variable selection procedure based on the Akaike Information Criterion (AIC) combined with a bootstrap resampling method. The final model was composed of the variables that were selected in at least 80% of the bootstrap samples; (c) the relevance of each variable was estimated in the final model. For this purpose, for each included variable, we refitted the model by permuting the variable values and we assessed the performance of this new model in terms of percentage root mean square error (RMSE). The worst the performance of the new model with respect to the original one, the more important the variable. This procedure was repeated 1,000 times.

Third, we compared the mortality risk of NH residents according to the eGFR categories also adjusting for potential confounders using Cox regression models. Potential confounders were selected through a backward stepwise regression procedure among the following variables: age, gender, ADL, nutritional status, GDS, MMSE, and the number of medications and diagnoses. The time from enrolment visit through the day of death was used as the time to failure variable for the model, and NH survivors were censored on the day of the last follow-up visit. The performance of these models was assessed by computing the cross-validated, 10-fold concordance index (37).

Finally, confusion matrices were used to determine the extent of agreement in CKD staging obtained by using the three different eGFR equations. The agreement was assessed with Cohen’s kappa without weighing. Survival analyses were carried out to evaluate whether the BIS1 and FAS equations improved risk reclassification for all-cause mortality when compared with the CKD-EPI equation. The attrition bias was finally investigated by age- and sex-adjusted logistic regression analysis of the three eGFR equations to loss to follow-up.

## Results

General and clinical characteristics of the NH residents are reported in Table 1. The mean age of these patients was 80.7 years, and 31.2% of them were men. After the follow-up period, 135 NH residents (25.9%) died, while the remaining 387 were still alive (74.1%). Approximately two-thirds of enrolled patients had at least 1 BADL dependency at the time of admission. Among the 195 patients without BADL disabilities, 89% were suffering from two or more chronic diseases, 60% were taking 5 or more drugs, and 15% were malnourished. Despite this, they did not have sufficient home support to guarantee the correct management of these problems and for these reasons they were admitted to NH.

**TABLE 1:** General and clinical characteristics of the NH residents recruited in the present study.

	Men (*N* = 163)	Women (*N* = 359)	Total (*N* = 522)
Age, years	80.3 ± 7.5	81.0 ± 8.0	80.7 ± 7.8
BMI (kg/m^2^)	25.8 ± 5.8	27.4 ± 6.5	26.9 ± 6.3
Dependency in 1 or more ADL (%)	100 (61.3)	227 (63.4)	327 (62.8)
SF-MNA < 12 (%)	53 (27.5)	136 (37.9)	189 (36.2)
MMSE	20.4 ± 6.3	18.5 ± 6.6	19.1 ± 6.5
Cognitive impairment (MMSE < 24)	83 (50.9)	221 (61.6)	304 (58.2)
GDS	4.5 ± 3.6	5.9 ± 4.0	5.5 ± 3.9
Depression (GDS > 5)	35 (21.5)	110 (30.6)	145 (27.8)
Hypertension (%)	119 (73.0)	262 (73.0)	381 (73.0)
Diabetes (%)	45 (27.6)	91 (25.3)	136 (26.1)
COPD (%)	45 (27.6)	56 (15.6)	101 (19.3)
Heart failure (%)	15 (9.2)	18 (5.0)	33 (6.3)
Ischemic heart disease (%)	49 (30.1)	81 (22.6)	130 (24.9)
Stroke (%)	21 (12.9)	33 (9.2)	54 (10.3)
CIRS score	14.8 ± 11.6	15.0 ± 13.0	15.0 ± 12.6
Number of medications	7.6 ± 3.9	7.2 ± 3.5	7.3 ± 3.6
Serum creatinine (mg/dL)	1.24 ± 0.44	1.06 ± 0.48	1.11 ± 0.47
eGFR BIS1 (ml/min/1.73 m^2^)	52.9 ± 19.0	49.9 ± 15.0	50.8 ± 16.4
eGFR FAS (ml/min/1.73 m^2^)	53.6 ± 23.2	48.9 ± 16.8	50.4 ± 19.2
eGFR CKD-EPI (ml/min/1.73 m^2^)	59.5 ± 18.7	55.9 ± 17.7	57.0 ± 18.0

*NH, nursing home.*

**FIGURE 1:**
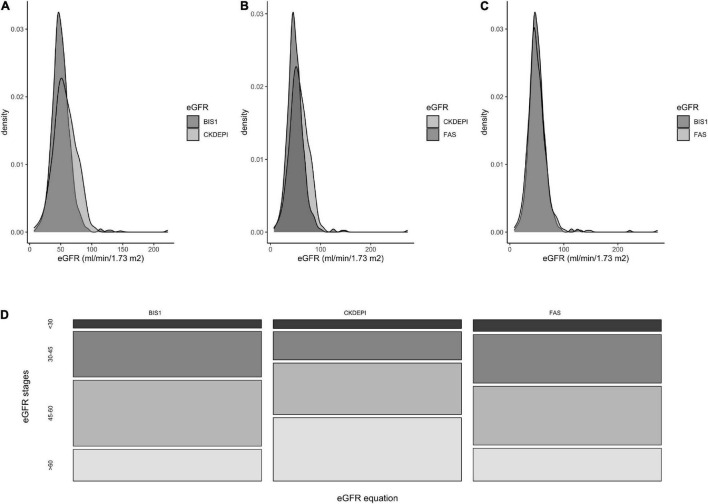
Frequencies distribution of eGFR values according to three different creatinine-based equations. **(A)** comparison between BIS1 and CKD-EPI equations, **(B)** between CKD-EPI and FAS, **(C)** between FAS and BIS1. **(D)** Distribution of NH residents according to the eGFR stages obtained by three different equations.

Figures 1A–C shows the frequency distribution of eGFR values according to the creatinine-based equations and the distribution of NH residents into categories based on eGFRs calculated using the adopted equation.

The percentage of residents with eGFR < 60 ml/min/1.73 m^2^ differed significantly when eGFR was calculated using the BIS1 (79.1%) or FAS (78.4%) compared with CKD-EPI (58.2%) equation (*p* < 0.001); conversely, the proportion of patients with eGFR < 30 ml/min/1.73 m^2^ did not show any significant difference in the comparison among equations and ranged between 5.6 and 7.7% (Figure 1D).

### Comparison Between Estimated Glomerular Filtration Rate Equations

**FIGURE 2:**
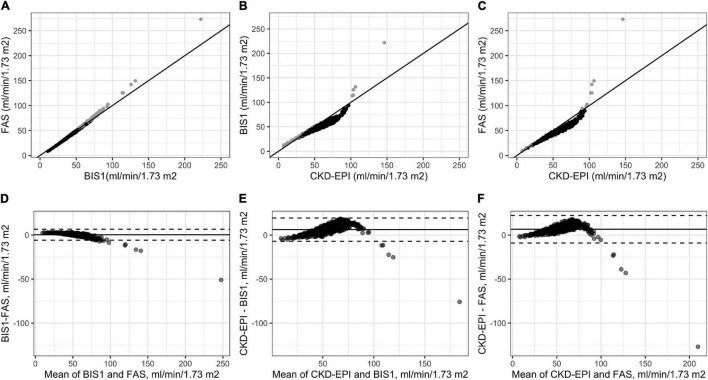
Correlations among the three adopted eGFR equations **(A–C)** and Bland–Altman analysis **(D–F)**. Gray points in **(A–C)** indicate NH residents for which the difference between the estimates of GFR through the equation reported on the *y*-axis is higher than those reported on the *x*-axis values. Gray points in (**A–C)** indicate NH residents for which the estimates of eGFR through the equation reported on the *y*-axis are higher than those reported on the *x*-axis; black points indicate NH residents for which the estimates of GFR through the equation reported on the *y*-axis is lower than those reported on the *x*-axis.

The three eGFR equations were strongly correlated with each other, even if the relationships between CKD-EPI and BIS or FAS were less linear with respect to that observed between BIS and FAS (Figures 2A–C).

Bland–Altman plots were generated to analyze the difference between CKD-EPI-BIS, CKD-EPI-FAS, and BIS-FAS against the mean of the two estimates, respectively, on the whole cohort of NH residents.

The Bland–Altman analysis showed that the bias between BIS and FAS was extremely low (0.45 ml/min/1.73 m^2^); a greater difference was observed only for patients with high eGFR values (Figure 2D). Conversely, a significant difference in calculated GFR values between CKD-EPI and BIS and also between CKD-EPI and FAS (6.21 ml/min/1.73 m^2^ and 6.65 ml/min/1.73 m^2^, respectively) was observed, peaking around 60 ml/min/1.73 m^2^ for both equations (CKD-EPI compared with BIS and CKD-EPI compared with FAS). Furthermore, the 95% upper limits of agreement were 19.40 and 22.36 ml/min/1.73 m^2^, respectively (Figures 2E,F).

**FIGURE 3:**
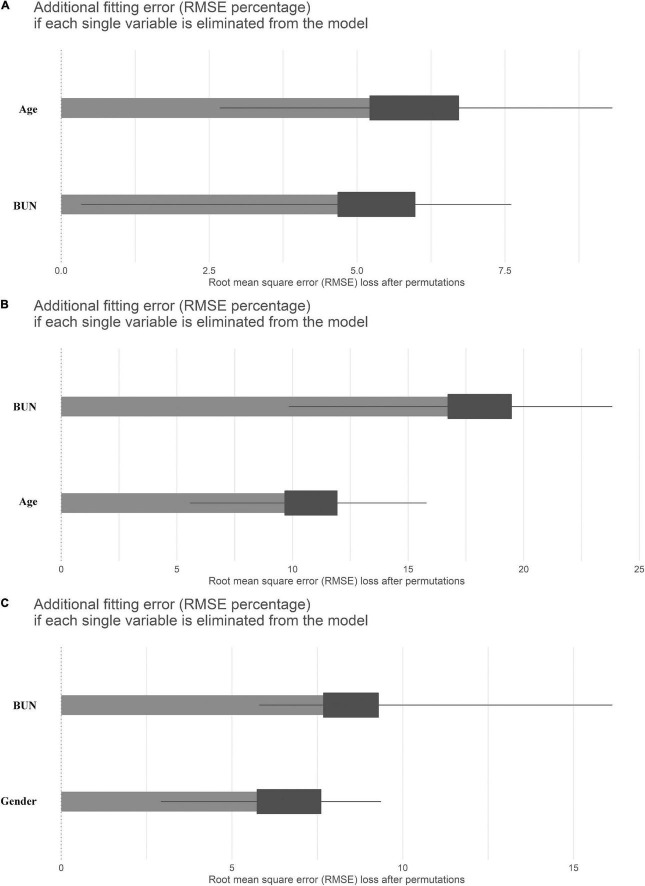
Means (over 1,000 permutations) of permutation-based variable-importance measures for the explanatory variables included in the model using root mean square error (RMSE) as the loss function. **(A)** Comparison between full age spectrum (FAS) and Chronic Kidney Disease Epidemiology Collaboration (CKD-EPI) equations, **(B)** between Berlin initiative study 1 (BIS1) and CKD-EPI, and **(C)** between FAS and BIS1.

We then investigated which factors affected the differences across the three eGFR estimation methods using a stepwise variable selection procedure combined with a bootstrap resampling method (Figure 3). Regarding the comparison between FAS and CKD-EPI (Figure 3A), the most important variable in the model was *age* followed by *BUN*. These results were paralleled by the results for the comparison between BIS1 and CKD-EPI (Figure 3B). In fact, once again the most important variables in the model were *BUN* and *age*. Regarding the comparison between FAS and BIS1 (Figure 3C), the most important variable in the model was *BUN*, followed by *gender*. The other variables selected by the univariate procedure were not relevant in terms of variable importance.

### Association Between Estimated Glomerular Filtration Rate and Mortality

**FIGURE 4:**
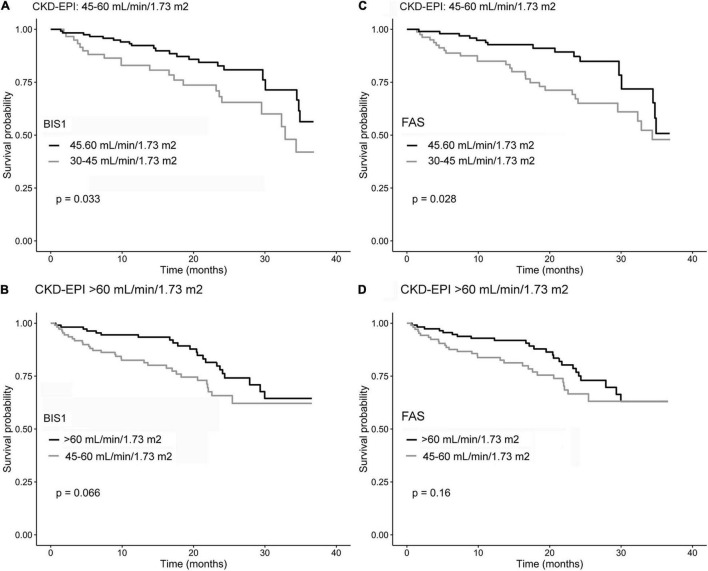
Kaplan–Meier survival curves of NH residents reclassified by comparing CKD-EPI and BIS1 **(A,B)** and CKD-EPI and FAS **(C,D)**. Gray curves indicate reclassified residents, while black curves indicate residents not reclassified.

Kaplan–Meier survival curves according to the eGFR categories are reported in Figure 4. Subjects with an eGFR < 30 ml/min/1.73 m^2^ exhibit the lowest survival probability during the follow-up time (*p*-value < 0.001 in all cases).

Cox regression analysis confirmed that this significant association also after adjusting for age at the recruitment and sex in Model 2 and additional confounders in Model 3 independently forms the adopted equations (Table 2). Other significant confounders associated with the outcome in Model 3 were age (*p* < 0.005), gender (*p* < 0.001), MNA < 12 (*p* < 0.008), ADL limitations (*p* < 0.028), and CIRS score (*p* < 0.001).

**TABLE 2:** The hazard ratio for the relationship between eGFR and survival chance during the follow-up time.

	Model 1			Model 2			Model 3		
	HR	95%CI	*P*-value	HR	95%CI	*P*-value	HR	95%CI	*P*-value
**BIS1**									
eGFR < 30	4.28	2.29–8.00	<0.001	2.01	1.04–3.88	0.038	3.82	1.64–8.85	0.002
eGFR 30-45	1.38	0.82–2.31	0.223	0.82	0.48–1.40	0.474	1.69	0.81–3.53	0.160
eGFR 45-60	1.30	0.79–2.14	0.289	1.07	0.65–1.77	0.783	2.00	0.98–4.05	0.056
**FAS**									
eGFR < 30	3.14	1.76–5.60	<0.001	1.64	0.89–3.00	0.112	2.35	1.10–5.03	0.028
eGFR 30-45	1.27	0.77–2.08	0.354	0.80	0.48–1.34	0.396	1.44	0.73–2.84	0.297
eGFR 45-60	1.14	0.70–1.87	0.600	0.95	0.58–1.57	0.848	1.55	0.79–3.04	0.199
**CDK-EPI**									
eGFR < 30	3.07	1.80–5.24	<0.001	1.93	1.12–3.34	0.019	2.37	1.27–4.41	0.007
eGFR 30-45	0.84	0.51–1.39	0.496	0.61	0.37–1.03	0.063	0.83	0.45–1.53	0.550
eGFR 45-60	0.97	0.65–1.45	0.891	0.87	0.58–1.30	0.482	1.48	0.90–2.45	0.126

*Model 1, unadjusted model.*

*Model 2, adjusted for age at the recruitment and sex.*

*Model 3, adjusted for age at the recruitment, sex, ADL, nutritional status, and CIRS score.*

*HR, hazard ratio; CI, confidence interval; eGFR, estimated glomerular filtration rate; ADL, activity of daily living; CIRS, Cumulative Illness Rating Score.*

However, while for the CDK-EPI and FAS, the HR estimates for the NH residents belonging to eGFR < 30 ml/min/1.73 m^2^ stage were approximately 2.4, for the BIS1 equation, the corresponding HR was approximately 3.8. Nevertheless, the accuracy in predicting overall mortality did not change significantly across equations in terms of cross-validated concordance index (BIS1 = 0.794, 95% CI: 0.740–0.849; FAS = 0.790, 95% CI: 0.738–0.843; CDK-EPI = 0.790; 95% CI: 0.736–0.845).

### Chronic Kidney Disease Stage Reclassification by Estimated Glomerular Filtration Rate Categories

Chronic Kidney Disease Epidemiology Collaboration showed a low level of agreement with BIS1 (66.9%) and FAS (62.6%) (Tables 3A,B). Of the 173 NH residents who were reclassified by BIS1 compared with CKD-EPI, the vast majority of reclassification (98.3%) placed NH residents in a higher-severity stage (lower eGFR category) and 1.7% to a lower CKD stage (higher eGFR category) (Table 3A). When comparing CKD-EPI and FAS, the 195 reclassified NH-residents by FAS were exclusively placed in a higher-severity CKD stage (Table 3B). At variance, BIS1 and FAS had a high level of agreement of approximately 92.7% (Table 3C).

**TABLE 3:** Confusion matrices of NH residents classified by either BIS1 or FAS compared with CKD-EPI equations.

A					
		**CKD-EPI-eGFR**			
		
		**<30**	**30–45**	**45–60**	**>60**

BIS1-eGFR	**<30**	**27**	2	0	0
	**30–45**	3	**95**	59	0
	**45–60**	0	0	**118**	109
	**>60**	0	0	0	**109**

**B**					

		**CKD-EPI-eGFR**			
		
		**<30**	**30–45**	**45–60**	**>60**

FAS-eGFR	<30	**30**	10	0	0
	30–45	0	**87**	80	0
	45–60	0	0	**97**	105
	>60	0	0	0	**113**

**C**					

		**FAS-eGFR**			
		
		**<30**	**30–45**	**45–60**	**>60**

BIS1-eGFR	<30	**29**	0	0	0
	30–45	11	**146**	0	0
	45–60	0	21	**201**	5
	>60	0	0	1	**108**

*NH, nursing home; BIS1, Berlin initiative study 1; FAS, full age spectrum; CKD-EPI, Chronic Kidney Disease Epidemiology Collaboration. Numbers in bold indicates NH residents classified in the same eGFR stage.*

**FIGURE 5:**
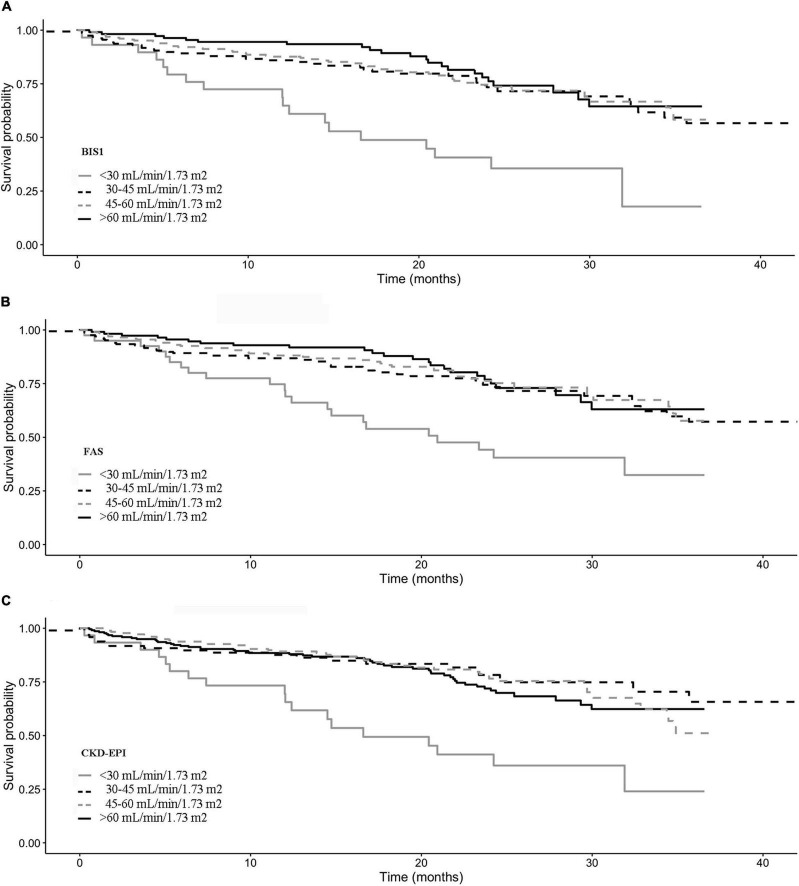
Kaplan–Meier survival curves according to the eGFR categories for **(A)** BIS1, **(B)** FAS, and **(C)** CDK-EPI.

The impact of reclassification on mortality estimates is reported in Figure 5. Among NH residents with CKD-EPI eGFR = 45–60 ml/min/1.73 m^2^, reclassification by BIS1 or FAS in the eGFR = 30–45 ml/min/1.73 m^2^ group was associated with significantly reduced survival. Among patients with CKD-EPI > 60 ml/min/1.73 m^2^, reclassification by BIS1 or FAS in the eGFR = 45–60 ml/min/1.73 m^2^ group was associated with a borderline significant reduced survival. Finally, attrition bias did not affect study results (*p* > 0.05 for all eGFR equations).

## Discussion

The present study shows that BIS1, FAS, and CKD-EPI equations could be used interchangeably only to some extent when staging CKD among older NH residents. Indeed, while BIS1 and FAS equations provide very similar estimates, CKD-EPI tends to overestimate values obtained with the other equations. Additionally, age, sex, and BUN are the main determinants of difference across equations. Finally, even if all three equations may predict prognosis with similar accuracy, NH residents with CKD-EPI = 45–60 ml/min/1.73 m^2^ reclassified by BIS1 or FAS in the 30–45 ml/min/1.73 m^2^ stage showed reduced survival compared to not reclassified ones.

Disagreement among eGFR values obtained with different equations has been frequently reported, but only few studies included the most recently introduced BIS and FAS equations, and data about older NH residents are distinctively lacking. Bland–Altman analysis carried out in the present study showed that the difference between CKD-EPI and BIS1 or FAS was very similar to that observed among community-dwelling individuals in former studies (38), as well as the negligible average difference between BIS and FAS is known to be related to the fact that FAS has been designed to match the BIS equation for ages > 70 years (30). Additionally, FAS was recently reported to predict eGFR calculated using the creatinine/cystatin C-based CKD-EPI equation with a median bias of 10.2 ml/min/1.73 m^2^ (95% CI = 9.2–10.9) in a population of 1,913 Chinese older patients with CKD (39). Such differences were observed despite the good performance of CKD-EPI, BIS, and FAS equations for measured GFR prediction. Similarly, a recent study by da Silva Selistre et al. (40) has shown that the median difference between CKD-EPI and FAS measured GFR was approximately −2.0 ml/min/1.73 m^2^, and the corresponding figure for the difference between CKD-EPI and BIS was nearly 0.0. Thus, considering this negligible difference between equations with respect to gold-standard measured GFR, the average differences between CKD-EPI and either BIS or FAS equations observed in our study would be unexpected. However, our study population is very different from that used to develop the CKD-EPI equation which included a pooled population with a wide age range (50 ± 15 years), but only 13.0% of people aged 65 years or above (36). At variance, the FAS equation was developed in a life-span perspective to allow eGFR calculation from childhood to older age (29), while the BIS equation was specifically developed in a population of people aged 70 or above (41). Thus, our findings further suggest that eGFR equations should be chosen taking into consideration the populations they had been developed for.

As regards, potential sources of discrepancy among eGFR equations, serum creatinine, and muscle mass were recently reported to be relevant correlates of disagreement, also impacting known sex differences of the CKD-EPI and BIS or FAS equations (42). While muscle mass was not assessed in the present study, our findings add to current knowledge by showing that BUN may also be involved in determining differences among equations in a population of older NH residents. The role of BUN was formerly investigated in a retrospective study of 2,685 hospitalized patients aged 58.7 ± 14.6 years where higher BUN levels were found associated with more disagreement in CKD staging between Modification of Diet in Renal Disease (MDRD) and CKD-EPI study equations (43). Additionally, lean body mass was formerly found to be the strongest predictor of deviations between eGFR vs. mean of creatinine and urea clearance (44), thus suggesting that the impact of BUN on eGFR discrepancies observed in our study may partly represent an indirect confirmation of the previously observed role of muscle mass.

Although the average difference among equations seems to be small, CKD stages may be significantly affected by equations used at the individual level. Indeed, 33.3% of patients staged as eGFR = 45–60 ml/min/1.73 m^2^ by CKD-EPI are classified as eGFR = 30–45 ml/min/1.73 m^2^ with BIS1, and the corresponding figure obtained with FAS is 45.2% in the present study. From the clinical point of view, failure to correctly classify older patients with CKD poses significant challenges, especially when dealing with decision-making about nephrology referral or managing kidney-cleared medications among older patients with multiple chronic diseases treated with polypharmacy regimens. Indeed, several disease-specific guideline recommendations suggest careful dosing of several drugs in patients with eGFR < 60 ml/min/1.73 m^2^ (45–47), and disagreement between equations may have important implications in terms of missing contraindication or dose reduction recommendation on one side, and underuse or underdosing on the other side. Prognostic implications also deserve to be mentioned. Although the eGFR study equations had comparable prognostic accuracy, BIS and FAS were able to reclassify NH residents, pertaining to a low-risk group with CKD-EPI. This finding suggests that the notion that eGFR estimated through BIS or FAS equations may slightly improve the discriminative capacity of CKD-EPI with respect to overall mortality in older community-dwelling subjects (48) could be extended to a frail population of older NH residents and implies that clinicians and epidemiologists should take into consideration equation-specific thresholds of risk in prognostic assessment and longitudinal studies involving such a frail population. Accordingly, it might be reductive to consider a given eGFR value indicative of a worse prognosis in the elderly without considering the estimating equation used.

The limitations of our study deserve to be mentioned. We used a limited set of data in our study, and we cannot exclude that the study equations may be differently sensitive to not measured factors, such as hydration status and body composition. Indeed, sarcopenia is known to impact creatinine production, and muscle mass may affect differences among creatinine-based eGFR equations (49). Given that body composition was not assessed in our study, further comparative analysis including extended datasets in these kinds of patients seems desirable. Since measured GFR was not available in our study, it seems advisable to consider the prognostic capacity as a property of the estimating equation only in part related to its validity and strictly dependent on the studied population. Additionally, eGFR calculation was based on a single measurement of serum creatinine, thus, we could not account for eGFR changes over time in our study. It should also be observed that CKD definition includes both GFR and albuminuria (50), and albuminuria was found to have the autonomous prognostic capacity in selected populations (51). Accordingly, considering also the albuminuria in the multivariable predictive model might have improved the definition of the prognostic capacity of kidney function measures. However, eGFR can be easily derived from routine blood analysis, whereas albuminuria cannot be considered a routine analysis and was not available in our dataset. Finally, cystatin C or other biomarkers of kidney function were not measured, and we cannot rule out that using different equations including other biomarkers may yield different results. However, using cystatin C-based rather than creatinine-based equations only marginally improves the concordance between CKD-EPI and BIS equations in a former study of community-dwelling older people (38).

In conclusion, our results show that CKD-EPI and BIS1 or FAS equations cannot be considered interchangeable to assess eGFR in a population of older NH residents. The observed differences are mainly explained by sex and BUN and could have a clinically relevant impact on diagnostic and therapeutic approaches. Additionally, BIS1 and FAS are able to identify a not negligible proportion of older NH residents carrying an increased risk of death even if classified as low risk by CKD-EPI. While our study does not allow to draw a definitive conclusion on the diagnostic accuracy of each individual equation, BIS and FAS equations provided very similar eGFR values and classification, and our findings suggest that these two equations specifically developed in older patients may be very useful for clinical assessment of eGFR in NH residents. Their substantial overlap would minimize discrepancy issues when monitoring the progression of CKD or prescribing/dosing kidney cleared medication.

## Data Availability Statement

The data that support the findings of this study are available from the corresponding author upon reasonable request.

## Ethics Statement

The studies involving human participants were reviewed and approved by the Regional Ethical Committee, Catanzaro (Prot. CE 119/2016). The patients/participants provided their written informed consent to participate in this study.

## Author Contributions

AM and GP: study design. EP, FM, ACoz, SC, and SG: data collection. AM, VL, and LS: data analyses. EP, AM, GP, and ACor: initial draft of the manuscript. All authors: finalize the manuscript. All authors contributed to the article and approved the submitted version.

## Conflict of Interest

SC and FM were employed by SADEL S.p.A. The remaining authors declare that the research was conducted in the absence of any commercial or financial relationships that could be construed as a potential conflict of interest.

## Publisher’s Note

All claims expressed in this article are solely those of the authors and do not necessarily represent those of their affiliated organizations, or those of the publisher, the editors and the reviewers. Any product that may be evaluated in this article, or claim that may be made by its manufacturer, is not guaranteed or endorsed by the publisher.

## References

[1] Hoffmann F Boeschen D Dörks M Herget-Rosenthal S Petersen J Schmiemann G. Renal insufficiency and medication in nursing home residents: a cross-sectional study (IMREN). *Dtsch Arztebl Int.* (2016) 113:92. 10.3238/arztebl.2016.0092 26931625 PMC4782265

[2] Aguilar EA Ashraf H Frontini M Ruiz M Reske TM Cefalu C. An analysis of chronic kidney disease risk factors in a Louisiana nursing home population: a cross-sectional study. *J La State Med Soc.* (2013) 165:260–3. 24350526

[3] Chan TC Yap DY Shea YF Luk KH Chan HW Chu LW. Prevalence and associated comorbidities of moderate to severe chronic renal impairment in Chinese nursing home older adults. *J Am Med Dir Assoc.* (2012) 13:630–3. 10.1016/j.jamda.2012.05.007 22698953

[4] Joseph J Koka M Aronow WS. Prevalence of moderate and severe renal insufficiency in older persons with hypertension, diabetes mellitus, coronary artery disease, peripheral arterial disease, ischemic stroke, or congestive heart failure in an academic nursing home. *J Am Med Dir Assoc.* (2008) 9:257–9. 10.1016/j.jamda.2008.01.002 18457801

[5] Schnelle J Osterweil D Globe D Sciarra A Audhya P Barlev A. Chronic kidney disease, anemia, and the association between chronic kidney disease-related anemia and activities of daily living in older nursing home residents. *J Am Med Dir Assoc.* (2009) 10:120–6. 10.1016/j.jamda.2008.08.012 19187880

[6] Garg AX Papaioannou A Ferko N Campbell G Clarke JA Ray JG. Estimating the prevalence of renal insufficiency in seniors requiring long-term care. *Kidney Int.* (2004) 65:649–53. 10.1111/j.1523-1755.2004.00412.x 14717937

[7] Robinson B Artz AS Culleton B Critchlow C Sciarra A Audhya P. Prevalence of anemia in the nursing home: contribution of chronic kidney disease. *J Am Geriatr Soc.* (2007) 55:1566–70. 10.1111/j.1532-5415.2007.01389.x 17727646

[8] Perazella MA Nolin TD. Adverse drug effects in patients with CKD: primum non nocere. *Clin J Am Soc Nephrol.* (2020) 15:1075–7. 10.2215/CJN.08890620 32611663 PMC7409750

[9] Matsushita K Mahmoodi BK Woodward M Emberson JR Jafar TH Jee SH Comparison of risk prediction using the CKD-EPI equation and the MDRD study equation for estimated glomerular filtration rate. *JAMA.* (2012) 307:1941–51. 10.1001/jama.2012.3954 22570462 PMC3837430

[10] Sarnak MJ Levey AS Schoolwerth AC Coresh J Culleton B Hamm LL Kidney disease as a risk factor for development of cardiovascular disease: a statement from the American heart association councils on kidney in cardiovascular disease, high blood pressure research, clinical cardiology, and epidemiology and prevention. *Hypertension.* (2003) 42:1050–65. 10.1161/01.HYP.0000102971.85504.7c 14604997

[11] Manjunath G Tighiouart H Coresh J Macleod B Salem DN Griffith JL Level of kidney function as a risk factor for cardiovascular outcomes in the elderly. *Kidney Int.* (2003) 63:1121–9. 10.1046/j.1523-1755.2003.00838.x 12631096

[12] Shlipak MG Sarnak MJ Katz R Fried LF Seliger SL Newman AB Cystatin c and the risk of death and cardiovascular events among elderly persons. *N Engl J Med.* (2005) 352:2049–60. 10.1056/NEJMoa043161 15901858

[13] Couser WG Remuzzi G Mendis S Tonelli M. The contribution of chronic kidney disease to the global burden of major noncommunicable diseases. *Kidney Int.* (2011) 80:1258–70. 10.1038/ki.2011.368 21993585

[14] Hallan S Astor B Romundstad S Aasarød K Kvenild K Coresh J. Association of kidney function and albuminuria with cardiovascular mortality in older vs younger individuals: the HUNT II study. *Arch Intern Med.* (2007) 167:2490–6. 10.1001/archinte.167.22.2490 18071172

[15] Corsonello A Freiberger E Lattanzio F. The Screening for Chronic Kidney Disease among Older People across Europe (Scope) Project: findings from Cross-Sectional Analysis. *BMC Geriatr.* (2020) 20:316. 10.1186/s12877-020-01701-w 33008358 PMC7531078

[16] Chadban SJ Briganti EM Kerr PG Dunstan DW Welborn TA Zimmet PZ Prevalence of kidney damage in Australian adults: the ausdiab kidney study. *J Am Soc Nephrol.* (2003) 14:S131–8. 10.1097/01.asn.0000070152.11927.4a 12819318

[17] Coresh J Astor BC Greene T Eknoyan G Levey AS. Prevalence of chronic kidney disease and decreased kidney function in the adult Us Population: third national health and nutrition examination survey. *Am J Kidney Dis.* (2003) 41:1–12. 10.1053/ajkd.2003.50007 12500213

[18] Bikbov B Purcell CA Levey AS Smith M Abdoli A Abebe M Global, regional, and national burden of chronic kidney disease, 1990–2017: a systematic analysis for the global burden of disease study 2017. *Lancet.* (2020) 395:709–33. 10.1016/S0140-6736(20)30045-3 32061315 PMC7049905

[19] Go DS Kim SH Park J Ryu DR Lee HJ Jo MW. Cost-utility analysis of the national health screening program for chronic kidney disease in Korea. *Nephrology.* (2019) 24:56–64. 10.1111/nep.13203 29206319

[20] Hallan SI Coresh J Astor BC Asberg A Powe NR Romundstad S International comparison of the relationship of chronic kidney disease prevalence and ESRD risk. *J Am Soc Nephrol.* (2006) 17:2275–84. 10.1681/asn.2005121273 16790511

[21] Komenda P Ferguson TW Macdonald K Rigatto C Koolage C Sood MM Cost-effectiveness of primary screening for CKD: a systematic review. *Am J Kidney Dis.* (2014) 63:789–97. 10.1053/j.ajkd.2013.12.012 24529536

[22] Rule AD Glassock RJ. GFR estimating equations: getting closer to the truth?. *Clin J Am Soc Nephrol.* (2013) 8:1414–20. 10.2215/cjn.01240213 23704300 PMC3731897

[23] KaiserMJBauerJMRamschCUterWGuigozYCederholmT Validation of the mini nutritional assessment short-form (MNA-SF): a practical tool for identification of nutritional status. *J Nutr Health Aging.* (2009) 13:782–8. 10.1007/s12603-009-0214-7 19812868

[24] Cox HJ Bhandari S Rigby AS Kilpatrick ES. Mortality at low and high estimated glomerular filtration rate values: a ‘U’ shaped curve. *Nephron Clin Pract.* (2008) 110:c67–72. 10.1159/000151720 18758185

[25] Tonelli M Klarenbach SW Lloyd AM James MT Bello AK Manns BJ Higher estimated glomerular filtration rates may be associated with increased risk of adverse outcomes, especially with concomitant proteinuria. *Kidney Int.* (2011) 80:1306–14. 10.1038/ki.2011.280 21849971

[26] Shastri S Katz R Rifkin DE Fried LF Odden MC Peralta CA Kidney function and mortality in octogenarians: cardiovascular health study all stars. *J Am Geriatr Soc.* (2012) 60:1201–7. 10.1111/j.1532-5415.2012.04046.x 22724391 PMC3902776

[27] Peters R Beckett N Poulter R Burch L Narkiewicz K Fagard R Kidney function in the very elderly with hypertension: data from the hypertension in the very elderly (HYVET) trial. *Age Ageing.* (2013) 42:253–8. 10.1093/ageing/afs109 22910302

[28] Montesanto A De Rango F Berardelli M Mari V Lattanzio F Passarino G Glomerular filtration rate in the elderly and in the oldest old: correlation with frailty and mortality. *Age.* (2014) 36:9641. 10.1007/s11357-014-9641-4 24664801 PMC4082598

[29] Schaeffner ES Ebert N Delanaye P Frei U Gaedeke J Jakob O Two novel equations to estimate kidney function in persons aged 70 years or older. *Ann Intern Med.* (2012) 157:471–81. 10.7326/0003-4819-157-7-201210020-00003 23027318

[30] Pottel H Hoste L Dubourg L Ebert N Schaeffner E Eriksen BO An Estimated Glomerular Filtration Rate Equation for the Full Age Spectrum. *Nephrol Dial Transplant.* (2016) 31:798–806. 10.1093/ndt/gfv454 26932693 PMC4848755

[31] Katz S Downs TD Cash HR Grotz RC. Progress in development of the index of ADL. *Gerontologist.* (1970) 10:20–30. 10.1093/geront/10.1_part_1.20 5420677

[32] Folstein MF Folstein SE McHugh PR. ”Mini-mental state”. A practical method for grading the cognitive state of patients for the clinician. *J Psychiatr Res.* (1975) 12:189–98. 10.1016/0022-3956(75)90026-6 1202204

[33] Lesher EL Berryhill JS. Validation of the geriatric depression scale–short form among inpatients. *J Clin Psychol.* (1994) 50:256–60. 10.1002/1097-4679(199403)50:23.0.co;2-e 8014251

[34] Vellas B Guigoz Y Garry PJ Nourhashemi F Bennahum D Lauque S The mini nutritional assessment (MNA) and its use in grading the nutritional state of elderly patients. *Nutrition.* (1999) 15:116–22. 10.1016/s0899-9007(98)00171-3 9990575

[35] Conwell Y Forbes NT Cox C Caine ED. Validation of a measure of physical illness burden at autopsy: the cumulative illness rating scale. *J Am Geriatr Soc.* (1993) 41:38–41. 10.1111/j.1532-5415.1993.tb05945.x 8418120

[36] Levey AS Stevens LA Schmid CH Zhang YL Castro AF III Feldman HI A new equation to estimate glomerular filtration rate. *Ann Intern Med.* (2009) 150:604–12. 10.7326/0003-4819-150-9-200905050-00006 19414839 PMC2763564

[37] Tsamardinos I Rakhshani A Lagani V. Performance-estimation properties of cross-validation-based protocols with simultaneous hyper-parameter optimization. *Int J Artif Intell Tools.* (2015) 24:1540023. 10.1142/S0218213015400230

[38] Corsonello A Pedone C Bandinelli S Ferrucci L Antonelli Incalzi R. Agreement between chronic kidney disease epidemiological collaboration and Berlin initiative study equations for estimating glomerular filtration rate in older people: the invecchiare in chianti (Aging in Chianti Region) study. *Geriatr Gerontol Int.* (2017) 17:1559–67. 10.1111/ggi.12932 27917582 PMC5804486

[39] Yan C Wu B Zeng M Yang G Ouyang C Zhang B Comparison of different equations for estimated glomerular filtration rate in Han Chinese patients with chronic kidney disease. *Clin Nephrol.* (2019) 91:301–10. 10.5414/cn109420 30802202

[40] da Silva Selistre L Rech DL de Souza V Iwaz J Lemoine S Dubourg L. Diagnostic performance of creatinine-based equations for estimating glomerular filtration rate in adults 65 years and older. *JAMA Intern Med.* (2019) 179:796–804. 10.1001/jamainternmed.2019.0223 31034005 PMC6547158

[41] Levey AS Inker LA. Assessment of glomerular filtration rate in health and disease: a state of the art review. *Clin Pharmacol Ther.* (2017) 102:405–19. 10.1002/cpt.729 28474735

[42] Corsonello A Roller-Wirnsberger R Wirnsberger G Ärnlöv J Carlsson AC Tap L Clinical implications of estimating glomerular filtration rate with three different equations among older people. preliminary results of the project “screening for chronic kidney disease among older people across Europe (Scope)”. *J Clin Med.* (2020) 9:294. 10.3390/jcm9020294 31973029 PMC7074235

[43] Lin SF Teng HE Lin HC. Blood urea nitrogen levels to verify estimated glomerular filtration rate, as derived from 2 commonly used equations. *Lab Med.* (2019) 50:298–305. 10.1093/labmed/lmz001 30892611

[44] Garcia-Naveiro R Rodriguez-Carmona A Pérez-Fontán M. Agreement between two routine methods of estimation of glomerular filtration rate in patients with advanced and terminal chronic renal failure. *Clin Nephrol.* (2005) 64:271–80. 10.5414/cnp64271 16240898

[45] Tuttle KR Bakris GL Bilous RW Chiang JL de Boer IH Goldstein-Fuchs J Diabetic kidney disease: a report from an ADA consensus conference. *Diabetes Care.* (2014) 37:2864–83. 10.2337/dc14-1296 25249672 PMC4170131

[46] Dowling TC Wang ES Ferrucci L Sorkin JD. Glomerular filtration rate equations overestimate creatinine clearance in older individuals enrolled in the baltimore longitudinal study on aging: impact on renal drug dosing. *Pharmacotherapy.* (2013) 33:912–21. 10.1002/phar.1282 23625813 PMC3732548

[47] Maccallum PK Mathur R Hull SA Saja K Green L Morris JK Patient safety and estimation of renal function in patients prescribed new oral anticoagulants for stroke prevention in atrial fibrillation: a cross-sectional study. *BMJ Open.* (2013) 3:e003343. 10.1136/bmjopen-2013-003343 24078751 PMC3787476

[48] Corsonello A Pedone C Bandinelli S Ferrucci L Antonelli Incalzi R. Predicting survival of older community-dwelling individuals according to five estimated glomerular filtration rate equations: the inchianti study. *Geriatr Gerontol Int.* (2018) 18:607–14. 10.1111/ggi.13225 29356245 PMC5891358

[49] Corsonello A Roller-Wirnsberger R Wirnsberger G Arnlov J Carlsson AC Tap L Clinical implications of estimating glomerular filtration rate with three different equations among older people. preliminary results of the project “screening for chronic kidney disease among older people across Europe (SCOPE)”. *J Clin Med.* (2020) 9:294. 10.3390/jcm9020294 PMC7074235 31973029

[50] Inker LA Astor BC Fox CH Isakova T Lash JP Peralta CA KDOQI US commentary on the 2012 kdigo clinical practice guideline for the evaluation and management of CKD. *Am J Kidney Dis.* (2014) 63:713–35. 10.1053/j.ajkd.2014.01.416 24647050

[51] Ford ES. Urinary albumin-creatinine ratio, estimated glomerular filtration rate, and all-cause mortality among US adults with obstructive lung function. *Chest.* (2015) 147:56–67. 10.1378/chest.13-2482 25079336 PMC4580968

